# Comparison of the Ability of Two Brands of CBCT with That of SEM to Detect the Marginal Leakage of Class V Composite Resin Restorations

**DOI:** 10.1155/2021/6688554

**Published:** 2021-06-30

**Authors:** Mitra Karbasi Kheir, Leili Khayam

**Affiliations:** ^1^Department of Oral and Maxillofacial Radiology, School of Dentistry, Islamic Azad University of Isfahan (Khorasgan), Isfahan, Iran; ^2^Department of Operative Dentistry, School of Dentistry, Islamic Azad University of Tabriz, Tabriz, Iran

## Abstract

**Objectives:**

This study was carried out to compare the ability of two common brands of cone-beam computed tomography (CBCT), including New Tom and Planmeca, to detect the marginal leakage of class V composite resins. The ability of each of the two brands of CBCT to detect the marginal leakage of class V composite resins was also compared with that of scanning electron microscopy (SEM).

**Methods:**

Class V cavities were prepared on the buccal surface of sixteen extracted caries-free human premolars. Cavities were conditioned and filled with composite resin. The teeth were immersed in 50% weight/weight aqueous silver nitrate solution for 24 hours. They were then taken out and rinsed with distilled water. Next, they were put in a developing solution. They were first viewed with New Tom and Planmeca CBCT units and were then sectioned and evaluated by an SEM.

**Results:**

The results of the Wilcoxon signed-rank test showed no significant difference between the mean marginal leakage scores of New Tom and Planmeca CBCT images (*p* value = 0.157) and between those of New Tom CBCT and SEM images (*p* value = 0.098). However, there was a significant difference between the mean marginal leakage scores of Planmeca CBCT and SEM images (*p* value = 0.023).

**Conclusion:**

There were no significant differences between New Tom and Planmeca CBCT units in the detection of marginal leakage of class V composite resins. However, when these CBCT units were compared with the SEM, the New Tom CBCT unit could detect the marginal leakage better than Planmeca.

## 1. Introduction

Marginal leakage in composite resin restorations results in undesirable consequences such as tooth sensitivity, recurrent caries, restoration margin discoloration, pulpal irritation, and eventually restoration failure [1–3]. Among different composite resin restorations, class V cavities have a relatively small configuration factor; consequently, the composite resins have less effective mechanical properties, and the bonding strength of the adhesive defines the outcome of the restoration. Hence, insufficient bond in class V composite resin cavities results in marginal leakage [1].

Various in vitro methods have been used to investigate the marginal leakage of composite resin restorations. These methods involve the use of biological, chemical, electrical, physical, and radioactive components. Among them, scanning electron microscopy (SEM) has proved to provide a better direct observation of the adaptation of a restorative material to the cavity margin [2]. Dye penetration methods are based on immersing the tooth sample in a dye solution and cutting the tooth through the center of the restoration to visualize the leakage of the restoration margins under a stereo microscope or an SEM. One disadvantage of these techniques is the loss of tooth structure integrity because of the need to cut the tooth to observe the leakage. Therefore, nondestructive radiographic methods have been introduced for the assessment of leakage.

The introduction of 3D radiographic methods with a small voxel size has helped the nondestructive detection of marginal leakage along the restoration margins. Two techniques have already been applied in this regard. The first one is microcomputed tomography (micro-CT), which is suggested for the assessment of marginal leakage in adhesive restorations and pit and fissure sealants. Another 3D technique in the field of dentistry is cone-beam computed tomography (CBCT), which has previously been used to detect small details like recurrent caries and vertical root fractures. It has recently been shown that CBCT can detect the marginal leakage of class V composite resin restorations in comparison to dye penetration methods [1, 4–8]. The increasing use of CBCT in dentistry to study fine details is undeniable.

There are various CBCT units with different features for diagnostic purposes. None of previous studies has compared the ability of different brands of CBCT units to detect the marginal leakage of class V composite resin restorations. Furthermore, none of them has compared CBCT with SEM regarding their ability to detect the marginal leakage of class V composite resin restorations, which is the gold standard. There are different brands of CBCT devices around the world. However, in the geographical area where the study was conducted, only two brands (New Tom and Planmeca) were mostly available. This study was designed to assess the ability of two brands of CBCT to detect the marginal leakage of class V composite resin restorations. The ability of each of the two brands of CBCT to detect the marginal leakage of class V composite resins was also compared with that of SEM.

## 2. Materials and Methods

### 2.1. Sample Selection

Sixteen caries-free human premolars were obtained from the patients who had their teeth extracted for orthodontic purposes. Patients' informed consent was obtained before the collection of samples. The teeth were cleaned of calculus, soft tissue, and debris by hand instrumentation after extraction. Then, they were stored in 0.5% chloramine-T solution in the refrigerator (4°C). It took three months to collect the extracted teeth. All the teeth were prepared and restored by a single operator to prevent interoperator bias.

### 2.2. Sample Preparation

A class V cavity was prepared on the buccal surface of each tooth by a cylindrical diamond bur (Tizkavan, Iran) mounted on a high-speed headpiece using air-water coolant spray (CH-4T5NSK B2/B3, Japan A1101800). Every four cavities were prepared by a new bur to avoid crack formation at the edges of the cavity. The cavities were checked by using a stereo microscope (Trinocular Zoom Stereo Microscope, SMP 200; HP, USA) to find probable cracks in the margin of cavities. The marginally cracked teeth were eliminated from the study to avoid false positive results due to the permeation of tracer through these spaces.

Class V cavities were 3 mm in width, 1.5 mm in depth, and 2.5 mm in height. The cavities were so prepared that the gingival margin of each cavity was extended beyond the cementoenamel junction (CEJ) onto the cementum, and the occlusal surface was limited to the enamel wall. The occlusal and gingival cavosurface margins of cavities were sharp and nonbeveled.

### 2.3. Composite Resin Filling

The cavities were conditioned with the Clearfil SE Bond adhesive system (SE: self-etching) (lot no. 71167; Kuraray Medical Inc., Okayama, Japan). The adhesive system was applied according to the manufacturers' instructions. The teeth were restored with a composite resin (GRADIA DIRECT, Anterior, CE0086, Japan). The composite restorative material was applied and condensed incrementally until the preparations were completely filled. Each increment was light-polymerized for 40 seconds prior to the placement of the subsequent increment using an LED light cure with 700 mW/cm^2^ light intensity (LED Turbo light cure; Taiwan). Then, the extra parts of restorations were removed by a polishing bur and disk.

### 2.4. Silver Nitrate Infiltration

The restored teeth were kept in distilled water for 24 hours at 37°C. To prevent the penetration of silver into areas other than the exposed margins, the tooth surfaces were sealed with two layers of nail varnish to be within approximately 1 mm of the restoration margins. The teeth were left at room temperature for one day to allow the nail varnish to dry. Then, they were immersed in 50% weight/weight aqueous silver nitrate solution for 24 hours in a dark place at room temperature. After that, the teeth were extracted and rinsed with distilled water. Next, they were put into a developing solution (Dental X-Ray Developer, KONIX; TURKUAZ, ISTANBUL, TURKEY) for eight hours and were then removed and abundantly rinsed with water. The teeth were cleaned using a toothbrush to remove silver depositions on the surfaces.

### 2.5. CBCT Imaging

**Figure 1:**
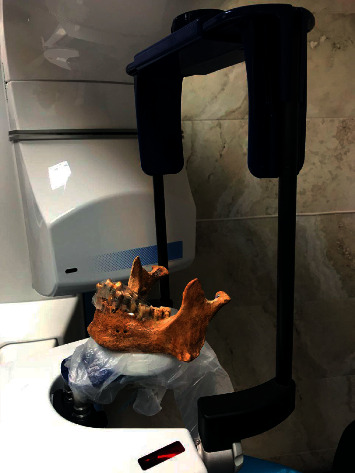
Teeth mounted on a dried mandible bone in the CBCT unit.

The teeth were mounted in the sockets of a human mandible bone and observed by CBCT. CBCT images were taken by two units, including New Tom CBCT unit (New Tom GiANO; Quantitative Radiology, Verona, Italy) and Planmeca CBCT unit (Planmeca ProMax 3D Mid machine; Helsinki, Finland) (Figure 1). The adjusted scan parameters of the New Tom CBCT unit were 90 kV, 3 mA, and 9 seconds. The field of view (FOV) was 8 *∗* 11 cm Hi-Res., and the voxel size was 75 *µ*m. The adjusted scan parameters of the Planmeca CBCT unit were 90 kV, 12.5 mA, and 15 seconds. The FOV was 10 *∗* 10 cm, and the voxel size was 150 *µ*m. The projections were analyzed by NNT (New Tom software) and Planmeca Romexis software in the cross-sectional plane (slice thickness = 0.5 mm and slice distance = 0.1 mm) by two maxillofacial radiologists to detect marginal leakage along the gingival and coronal walls of class V cavities. The observers were blind to each other's responses. No software was used for artifact reduction.

### 2.6. Scanning Electron Microscopy

After taking the CBCT projections, the teeth were mounted into a green self-curing acrylic resin and sectioned into two halves longitudinally in buccolingual direction by a tooth-sectioning device with a water-cooled diamond disk (Cutting machine; Fanavaran Pars Industrial CO, Iran). The sectioned teeth were evaluated by two observers for the penetration of silver nitrate using SEM (Cam-Scan MV2300; Slovakia) with 80–250x magnification and a digital camera (Moticam 480 Digital Camera, SP10.0224; Motic Instruments, Inc., USA). The observers were blind to each other's responses.

The marginal leakage along the gingival and coronal walls was evaluated according to Mousavinasab et al.'s study [9]:

  0: no leakage visible
  I: penetration of dye less than half of the gingival floor depth
  II: penetration of dye more than half of the gingival or occlusal floor depth without reaching the axial angle
  III: penetration of dye along the axial wall and toward the pulp

### 2.7. Statistical Analysis

The interobserver agreement was determined with Cohen's kappa coefficient. Because two observers analyzed every tooth in each method, the average response of the two observers was taken for comparisons. Statistical comparisons were made between the two brands of CBCT units and then between each CBCT unit and SEM to determine the leakage scores of each method. Since the data distribution was not normal, the Wilcoxon signed-rank test was used to compare the gingival wall leakage scores between the above two dependent groups. The data were analyzed by the Statistical Package for Social Sciences (SPSS) (version 22; SPSS Inc., Chicago, IL). The significance level was set at 0.05.

## 3. Results

The leakage scores of restorations in the gingival floor measured by each technique are presented in Table 1. Assessment of interobserver agreement for New Tom CBCT and Planmeca CBCT images showed a Cohen's kappa coefficient of 0.887 (*p* value = 0.001), and assessment of interobserver agreement for SEM images showed a Cohen's kappa coefficient of 1.00 (*p* value = 0.00).

**Figure 2:**
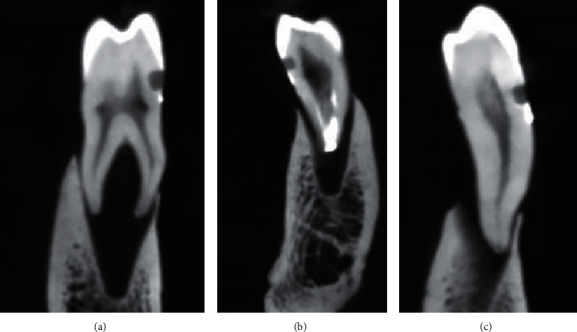
Marginal leakage on the gingival wall detected by the New Tom CBCT unit (New Tom GiANO; Quantitative Radiology, Verona, Italy). (a) Score 0. (b) Score 1. (c) Score 2.

**Figure 3:**
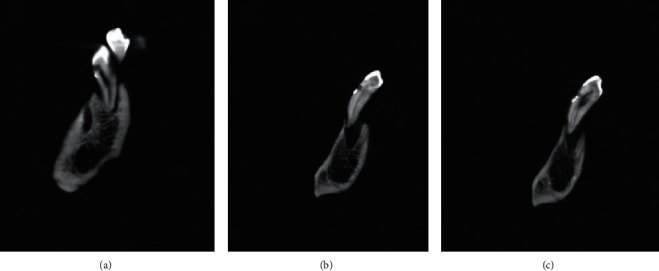
Marginal leakage on the gingival wall detected by the Planmeca CBCT unit (Planmeca ProMax 3D Mid machine, Helsinki, Finland). (a) Score 0. (b) Score 1. (c) Score 2.

**Figure 4:**
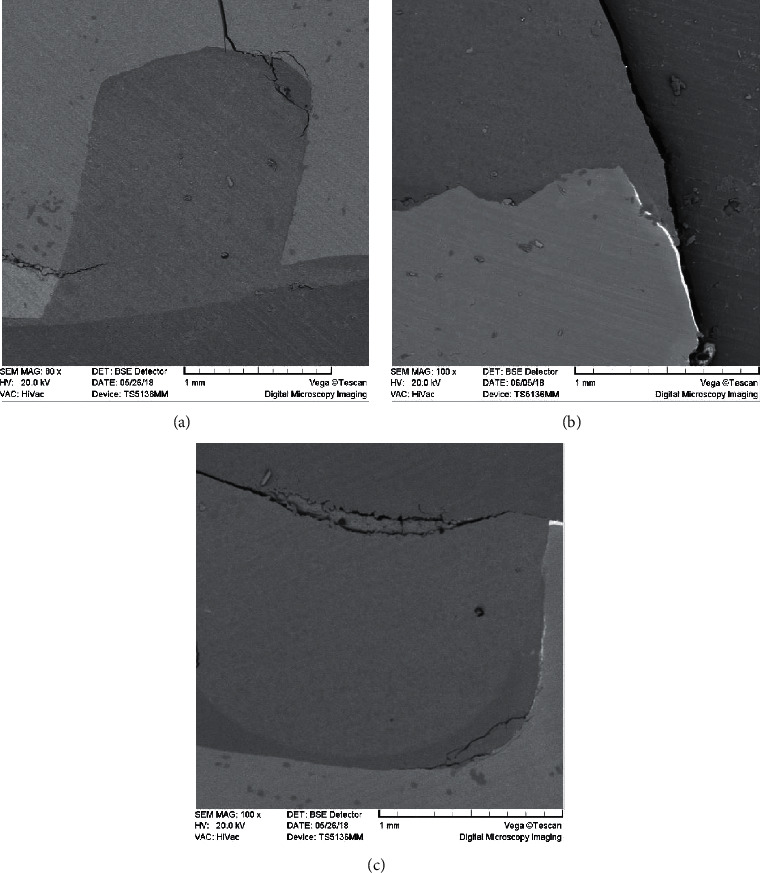
Marginal leakage on coronal and gingival walls detected by SEM (Cam-Scan MV2300; Slovakia). (a) Marginal leakage, score 0, on the coronal and gingival walls, 60x. (b) Marginal leakage, score 1, on the gingival wall, 100x. (c) Marginal leakage, score 2, on the gingival wall, 100x.

The results of the Wilcoxon signed-rank test showed no significant difference between the mean marginal leakage scores of New Tom and Planmeca CBCT images (*p* value = 0.157) and between the mean marginal leakage scores of New Tom CBCT and SEM images (*p* value = 0.098). However, the results of the Wilcoxon signed-rank test showed a significant difference between the mean marginal leakage scores of Planmeca CBCT and SEM images (*p* value = 0.023), as the marginal leakage scores of Planmeca CBCT were higher than those of SEM images (Tables 2–4). Both observers reported the same scores (0: no leakage visible) for the coronal walls in CBCT and SEM examinations. Figures 2–4 show the marginal leakage along the gingival and coronal walls of class V composite resin restorations using SEM and CBCT units.

## 4. Discussion

Marginal leakage is an adverse outcome caused by insufficient bond of composite resin restorations. This study was performed to assess the ability of two brands of CBCT units to detect the marginal leakage of class V composite resin restorations in comparison with SEM.

The results showed that CBCT could detect the marginal leakage of class V composite resin restorations. Although there were no statistically significant differences between the two brands of CBCT in the detection of marginal leakage, the comparison of each of these two brands with SEM showed different results. There was no significant difference between the marginal leakage scores of New Tom CBCT and SEM images, while there was a significant difference between the marginal leakage scores of Planmeca CBCT and SEM images. Indeed, the New Tom CBCT unit had better performance than the Planmeca CBCT unit in detecting marginal leakage. This better performance of the New Tom CBCT unit could be related to its smaller field of view (FOV) and the smaller voxel size. A smaller FOV reduces the scatter radiation, which degrades the image quality. The scatter radiation is recorded by the cone-beam area detector. This additional recording of X-ray beam is called noise. Therefore, the selection of a smaller FOV reduces the image noise and enhances its quality. Moreover, it has been proven that the presentation of details on a CBCT image strongly depends on spatial resolution (SR). SR is determined by the voxel size, and a smaller voxel size causes higher SR [10].

In this study, the smallest FOV was selected in both CBCT examinations to enhance the ability of observers to detect marginal leakage and provide similar conditions for the comparison of the two CBCT units. The smallest FOV was 8 *∗* 11 in the New Tom CBCT unit and 10 *∗* 10 in the Planmeca CBCT unit. Our results showed that even this small difference in the FOV between the two units affected the amount of noise and degraded the image quality. In other words, the larger FOV in the Planmeca CBCT unit was associated with more noise production, which reduced the image quality. Moreover, the New Tom CBCT unit had a smaller voxel size and higher SR than the Planmeca unit. The minimum voxel size was fixed in the two devices (75 *µ*m in New Tom and 150 *µ*m in Planmeca), and it was not possible to equalize the voxel size of both devices in this study. Therefore, the combination of higher SR and smaller FOV contributed to the better performance of the New Tom CBCT unit than Planmeca in the detection of marginal leakage.

This study assessed the marginal leakage of both coronal and gingival walls of class V composite resin restorations. None of the tooth samples showed marginal leakage on the coronal walls of class V composite resin restorations using SEM and CBCT. The coronal wall contains enamel, and the bond strength to enamel is more than that of dentin. Consequently, the marginal leakage of the coronal wall is less than that of the gingival floor. The results of this study were in agreement with those of other studies that used dye penetration and micro-CT methods to assess marginal leakage along coronal and gingival walls [1–4, 11, 12].

**Figure 5:**
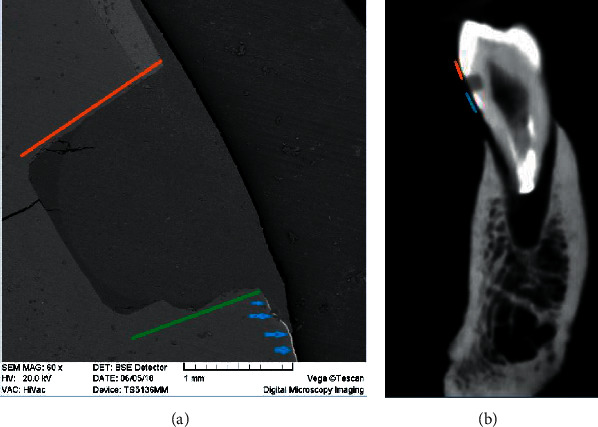
Silver nitrate leakage along gingival and coronal walls of a composite resin restoration. (a) Red line: coronal wall; green line: gingival wall; blue arrows: silver nitrate. (b) Red line: enamel radiopacity; blue line: silver nitrate radiopacity.

CBCT has limitations in assessing the marginal leakage of the coronal wall of class V cavities. As shown in Figure 5, SEM can reveal the marginal leakage of the coronal walls as well as the gingival walls of class V composite resin restorations. However, this might not be always true for the CBCT because some parts of the coronal wall of class V cavities are located in the enamel, and the radiopacity of enamel obscures the radiopacity of silver nitrate tracer. As a result, the CBCT observer may underestimate the amount of leakage on the coronal wall. However, when the leakage extends to the dentin beyond the enamel, it is definitively recognizable on the CBCT images. This can be considered one of the limitations of CBCT against SEM in the detection of marginal leakage.

We could not find any similar articles in the literature that compared the capability of CBCT and SEM in the detection of marginal leakage. The most similar one was the study of Khoroushi et al. which compared the ability of CBCT (New Tom VGi Evo) and stereo microscopes to detect the marginal leakage of class V composite resin restorations and found no significant difference between these two methods [5]. The present study is basically different from the above study in that it compared the ability of two CBCT units (NewTom GiANO and Planmeca) and SEM to detect marginal leakage. Since the use of stereo microscope for the detection of marginal leakage has become obsolete and has now been replaced by SEM, it seems necessary to compare the ability of CBCT and SEM to detect marginal leakage.

**Figure 6:**
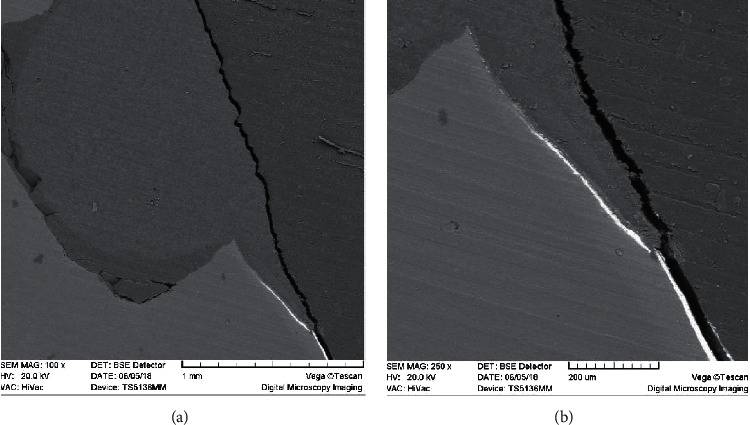
Marginal leakage of the gingival wall at different SEM magnifications. (a) 100x magnification. (b) 250x magnification.

As shown in Rengo et al. study on micro-CT, one of the advantages of SEM over CT is the ability of SEM in image magnification [1]. This study used 80–250x magnification to assess the leakage through SEM images. The entire tooth-restoration interface was quite visible at 80x magnification, and as the SEM magnification was increased above 80x, more details became visible and the silver nitrate tracing became more accurate (Figure 6). However, increasing the magnification of CBCT images degrades the ability to evaluate the details due to the loss of image clarity. This can be taken into account as another limitation of CBCT in the detection of leakage.

As a previous study has shown, the proximity of the radiopacity of the tracer to the gingival wall of the cavity can mislead the observers to underestimate the marginal leakage scores on the CBCT images [5]. In the present study, the observers who evaluated the CBCT images encountered this problem, too. However, SEM did not show such a limitation because zooming SEM images could depict this proximity very well and prevented misinterpretation.

Nevertheless, one advantage of CBCT over SEM is being nondestructive for tooth integrity. CBCT makes the evaluation of margins possible without tooth sectioning. Another advantage of CBCT is the ability to display the multiplanar images of the restoration margins and to provide the possibility of moving between sectional images in any plane along the restoration margin [5, 13].

Silver nitrate solution was used in this study as a tracer because of the chemical and radiographic properties of silver in leakage examinations. There is no agreement on the ideal concentration of silver nitrate and the optimal time of tooth immersion for CT observations. Yet, it appears that tooth immersion in 50% silver nitrate solution for 14–24 hours provides appropriate interface staining of silver ions and displays a good radiopaque contrast [1, 4, 14, 15].

One of the limitations of the present study was the lack of soft tissue simulation. Soft tissue produces some noises in the CBCT images, which degrades the image quality and may affect the interpretations [10]. Another limitation of this study was comparison of only the two brands NewTom and Planmeca for reasons beyond the choice of researchers. Considering the importance of FOV and voxel size in the detection of marginal leakage and wide variations of such components in different brands of CBCT, future studies are suggested to compare other CBCT brands with SEM.

## 5. Conclusion

Within the limitations of this study, it can be concluded that there were no significant differences between New Tom and Planmeca CBCT units in detecting the marginal leakage of class V composite resins. However, when these CBCT units were compared with the SEM, the New Tom CBCT unit could detect the marginal leakage better than Planmeca.

## Figures and Tables

**Table 1:** The scores of leakage along the gingival wall of restorations in CBCT units and SEM.

Tooth number	Observer 1 New Tom CBCT	Observer 2 New Tom CBCT	Observer 1 Planmeca CBCT	Observer 2 Planmeca CBCT	Observer 1 SEM	Observer 2 SEM
1	1	1	1	1	0	0
2	0	0	1	1	0	0
3	2	2	2	2	2	2
4	0	0	0	0	0	0
5	0	1	0	1	0	0
6	0	0	0	0	0	0
7	1	1	1	1	0	0
8	1	1	1	1	1	1
9	0	0	0	0	0	0
10	1	1	1	1	1	1
11	0	0	0	0	0	0
12	0	0	0	0	0	0
13	0	0	0	0	0	0
14	1	1	1	1	0	0
15	0	0	1	1	0	0
16	1	1	1	1	1	1

**Table 2:** Comparison of marginal leakage scores between Planmeca and New Tom CBCT units.

		*N*	Mean rank	Sum of ranks	*Z*	Asymp. sig. (2-tailed)
Planmeca-New Tom CBCT units	Negative ranks	0^a^	0.00	0.00	−1.414^b^	0.157
	Positive ranks	2^b^	1.50	3.00		
	Ties	14^c^				
	Total	16				

^a^Planmeca < New Tom. ^b^Planmeca > New Tom. ^c^Planmeca = New Tom.

**Table 3:** Comparison of marginal leakage scores between New Tom CBCT unit and SEM.

		*N*	Mean rank	Sum of ranks	*Z*	Asymp. sig. (2-tailed)
SEM-New Tom CBCT	Negative ranks	4^a^	3.38	13.50	−1.656^b^	0.098
	Positive ranks	1^b^	1.50	1.50		
	Ties	11^c^				
	Total	16				

^a^SEM < New Tom CBCT. ^b^SEM > New Tom CBCT. ^c^SEM = New Tom CBCT.

**Table 4:** Comparison of marginal leakage scores between Planmeca CBCT unit and SEM.

		*N*	Mean rank	Sum of ranks	*Z*	Asymp. sig. (2-tailed)
SEM-Planmeca CBCT	Negative ranks	6^a^	3.50	21.00	−2.271^b^	0.023
	Positive ranks	0^b^	0.00	0.00		
	Ties	10^c^				
	Total	16				

^a^SEM < Planmeca CBCT. ^b^SEM > Planmeca CBCT. ^c^SEM = Planmeca CBCT.

## Data Availability

The data used in the analysis of the present study can be provided by the corresponding author upon request.

## Abbreviations

SEM: Scanning electron microscopy
Micro-CT: Microcomputed tomography
CBCT: Cone-beam computed tomography
CT: Computed tomography
SR: Spatial resolution
USA: United States of America
CEJ: Cementoenamel junction
3D: Three-dimensional
˚C: Degree Celsius
mm: Millimeter
cm: Centimeter
*µ*m: Micrometer
SE: Self-etching
LED: Light-emitting diode
NSK: Machinery manufacturing company
mA: Milliampere
kV: Kilovoltage
mW: Megawatt
FOV: Field of view
Hi-Res: High resolution
NNT: New Tom software
Inc.: Incorporated
CO: Company or corporation
SPSS: Statistical Package for Social Sciences
IL: Illinois
*p* value: Calculated probability.
